# The Stability and Efficency of CPB Cells Were Acclimated for Virus Proliferation

**DOI:** 10.3390/vaccines12030220

**Published:** 2024-02-20

**Authors:** Yinjie Niu, Saiya Ma, Hongru Liang, Xiaozhe Fu, Baofu Ma, Qiang Lin, Xia Luo, Ningqiu Li

**Affiliations:** Pearl River Fisheries Research Institute, Chinese Academy of Fishery Sciences, Key Laboratory of Fishery Drug Development, Ministry of Agriculture and Rural Affairs, Guangdong Provincial Key Laboratory of Aquatic Animal Immunology and Sustainable Aquaculture, Guangzhou 510380, China; nyj@prfri.ac.cn (Y.N.); saiyama06152024@163.com (S.M.); hrliang@prfri.ac.cn (H.L.); fuxiaozhe@prfri.ac.cn (X.F.); mabf@prfri.ac.cn (B.M.); linq@prfri.ac.cn (Q.L.); luoxia@prfri.ac.cn (X.L.)

**Keywords:** CPB p300 cells, ISKNV, SCRV, LMBV, multiplication

## Abstract

Background: Vaccinations are still the most effective means of preventing and controlling fish viral diseases, and cells are an important substrate for the production of a viral vaccine. Therefore, the rapid-stable growth and virus sensitivity of cells are urgently needed. Methods: Chinese perch brain 100th passage (CPB p100) were acclimated in a low serum with 5% FBS L-15 for 50 passages, then transferred to 8% FBS L-15 for 150 passages. Additionally, the morphology and cell type of CPB 300th passage (CPB p300) cells were identified. We analyzed the transfection efficiency and virus sensitivity of CPB p300 cells, and then optimized the conditions of ISKNV, SCRV, and LMBV multiplication in CPB cells. Results: CPB p300 cells were more homogeneous, and the spread diameter (20–30) µm in CPB p300 cells became the dominant population. The doubling time of CPB p300 was 1.5 times shorter than that of CPB p100.However, multiplication rate of CPB p300 was 1.37 times higher than CPB p100. CPB p300 cells were susceptible to ISKNV, SCRV, and LMBV, and the optimal conditions of ISKNV, SCRV, and LMBV multiplication were simultaneous incubation, 0.6 × 10^5^ cells/cm^2^ and MOI = 0.1; infection at 48 h, 0.8 × 10^5^ cells/cm^2^ and MOI = 0.01; simultaneous incubation, 0.7 × 10^5^ cells/cm^2^ and MOI = 0.05, respectively. The time and economic costs of ISKNV, SCRV, and LMBV multiplication in CPB p300 cells were significantly reduced. Conclusions: The acquisition of CPB p300 cells laid a good material foundation for the production of ISKNV, SCRV, and LMBV vaccines.

## 1. Introduction

Mandarin fish (*Sinipercachuatsi*) and Largemouth bass (*Micropterus salmoides*) are important aquaculture species in Asian countries [1,2]. The mandarin fish and largemouth bass are not only economically important fish, but also an important part of food security. However, viral diseases outbreaks have frequently caused serious economic losses to *Siniperca chuatsi* and *Micropterus salmoides.* Infectious spleen and kidney necrosis virus disease (ISKNVD) and *Siniperca chuatsi* rhabdovirus disease (SCRVD) are major pathogens of the mandarin fish and largemouth bass farming industry [3,4,5,6,7]. ISKNV can infect different sizes of fish, whilst SCRV is a member of the genus Siniperhabdovirus in Rhabdoviridae [8]. Largemouth bass virus (LMBV) is the most serious disease affecting the largemouth bass industry [9,10]. At present, there are no effective prevention and control measures for those viral diseases. Successful vaccination is the most effective means of preventing and controlling viral diseases [11]. Vaccination has become the international routinely practice for the prevention and controlling fish diseases in aquaculture [12]. Research about fish vaccines started late in China. Xianle Yang et al. developed cell culture inactivated vaccines for grass carp reovirus (GCRV) in 1984, and obtained the first new veterinary medicine certificate for a fish vaccine in 1992. At present, one inactivated vaccine and one attenuated vaccine of GCRV, an inactivated vaccine for ISKNV, three fish viral vaccines have obtained Chinese new veterinary drug certificates. However, these vaccines have not yet been used on a large scale. The reason for this lies in small production scales, unstable quality of vaccines, and high production costs, which causes the backward vaccine production process. Cells are the basic material of vaccine production process [13]. 

Cells are used in a variety of applications in industry and research. Cells are an especially important substrate for the production of viral vaccines [14]. Therefore, the CPB (Chinese perch brain, CPB) cells were established by our laboratory from the brain of *Siniperca chuatsi* [15]. The CPB cells have been passaged more than 100 times. On the basis of the CPB cells line, our laboratory constructed an ISKNV and SCRV bivalent inactivated vaccine [16], and screened a SCRV potential live vaccine candidate for Chinese perch against SCRV disease [17]. For scale production of CPB cells for ISKNV and SCRV vaccines, we developed a suspension culture system of CPB cells on cytodex 1 microcarrier in 3-L bioreactor [18]. The stability, growth rate, and virus sensitivity of cells directly influence vaccine production scales, production costs, and competitiveness [19,20,21]. At present, CPB cells below 120 passages are prone to mutation, apoptosis, and are not stable. Compared with Vero and MDCK cells, the proliferative capacity of CPB cells was slightly weaker [22,23]. The instability and low productive capacity of CPB p100 cells influenced the development of the vaccine production process and hindered the marketing of ISKNV, SCRV, and LMBV vaccines.

In order to reduce vaccine production costs, and improve vaccines quality and market competitiveness, we cultured the CPB p100 cells in 5% FBS L-15 for 50 passages, then transferred to 8% FBS L-15 for 150 passages. The morphology and cell type of CPB p300 cells were identified. We analyzed the transfection efficiency of CPB p300 cells and the susceptibilities of ISKNV, SCRV, LMBV in CPB p300 cells, and optimized the multiplication conditions of three viruses. Taken together, we acclimated the high proliferation of CPB p300 cells and provided a solid material base for the production of ISKNV, SCRV and LMBV vaccines. 

## 2. Material and Methods

### 2.1. Cells and Virus Strains

The CPB cells were domesticated from Mandarin fish brain and stored in our laboratory [15]. The ISKNV QY-2009 and SCRV QY-2014 were isolated from the tissue of diseased *Siniperca chuatsi* and stored by our laboratory [3]. LBMV-2007 virus strains were isolated from the spleen of diseased *Microptencs salmoides* and kept in our lab. The Leibovitz’s L-15 medium, 0.25% trypsin-EDTA and phosphate-buffered saline (PBS) were purchased from Cytiva Hyclone (Logan, UT, USA).

### 2.2. Cells Acclimation

The CPB p100 CPB cells were cultured in a low serum culture medium (Leibovitz’s L-15 medium with 5% fetal bovine serum, 5% FBS L-15). When the cells were 90–100% confluence, the passage was performed. The briefly performed steps was as follows: the CPB cells in T25 flask were rinsed once with 10 mL PBS, and incubated in 1 mL 0.25% trypsin-EDTA for 4–5 min, then added10–15 mL 5% L-15 medium, blown, mixed well, and dispensed into T25 flask. If the cells fail to reach 90–100% confluence, the culture medium was changed every 5 days. CPB cells was passaged according to the above me cells were transferred to 8% FBS L-15 for continuous passage. 

### 2.3. Morphological Characteristics of CPB Cells 

CPB p100, p150, and p300 cells were cultured for 72 h, then 100 cells of CPB p100, p150, and p300 cells were randomly selected and their long diameter were measured, then fixed with 4% paraformaldehyde for 30 min, respectively. The indirect immunofluorescence of fixed cells were performed with cytokeratin 19 antibody (marker protein of epithelial cells), and then were observed with the fluorescent inverted microscope (OLYMPUS IX83, Olympus, Tokyo, Japan). 

### 2.4. The Growth Characteristics of CPB Cells

CPB p100, p150, and p300 cells were cultured in T75 flasks with the cell density of 2.0 × 10^5^ cell/mL, respectively. The cells were digested and counted at 12, 24, 48, 72, 96, 120, 144 and 168 h. The number of cells/mL was calculated and the growth curve of each passage was analyzed. The digestion time of CPB p100, p150, and p300 cells was measured.

### 2.5. Transfection Efficiency

CPB p100 and p300 cells were propagated in 12 well plate at a density of 2.5 × 10^5^ cell/mL, respectively. Cells at 80–90% confluence were transfected with 2 μgpEGFP eukaryotic expression vector. After 48 h of transfection, the EGFP expression was observed by fluorescent inverted microscope (OLIMPUS IX83)

### 2.6. Virus Susceptibility 

In total, 2.5 × 10^5^ of CPB p100 and p300 cells were seeded in T25. After 24 h cultivation, the cells were infected with ISKNV (MOI = 0.1), SCRV (MOI = 0.001), and LMBV (MOI = 0.01). Those cells were observed daily for the cytopathic effect of ISKNV, SCRV, and LMBV, and the viral titer were measured by 50% tissue culture infective dose (TCID_50_) in 96-well plates.

### 2.7. Virus Multiplication Conditions

In order to obtain higher ISKNV, SCRV, and LMBV titers: ISKNV and LMBV were incubated in cell culture at 0 h, 24 h, and 48 h, respectively. SCRV was incubated in cell culture at 24 h, 48 h, and 72 h, respectively. Then, 0.5 × 10^5^, 0.6 × 10^5^ and 0.7 × 10^5^ cells/cm^2^of p300 were infected with ISKNV(MOI = 0.1), respectively. 0.6 × 10^5^, 0.7 × 10^5^ and 0.8 × 10^5^ cells/cm^2^ of p300 were infected with LMBV (MOI = 0.01), respectively. 0.7 × 10^5^, 0.8 × 10^5^ and 0.9 × 10^5^ cells/cm^2^ of p100 and p300 cells were infected with SCRV (MOI = 0.001), respectively. Next, p300 cells were infected with ISKNV (MOI =1, 0.1, 0.01), LMBV (MOI = 0.5, 0.05, 0.005), and SCRV (MOI = 0.01, 0.001, 0.0001), respectively. We selected the optical cell density, placed cells in 6-well plates, and then was infected with ISKNV, SCRV and LMBV. The cells were frozen and thawed at 12, 24, 48, 72, 96, 120, and 144 h post- infection, and the cell debris was removed by centrifugation. The viral titers of different time points were measured by TCID_50_. 

### 2.8. The Time and Economic Cost of Viral Multiplication

We selected the optical cell density to spread in a T75 flask, then it was infected with ISKNV (synchronous infection, MOI = 0.1), SCRV (infection at 48 h, 0.001 and LMBV (synchronous infection, 0.05). The viral titer was measured by TCID_50_. The time cost was calculated as TCID_50_ × V (mL)/time (d). The economic cost is calculated as TCID_50_/total cost (Yuan).

### 2.9. Statistical Analyses

Results are expressed as the means ± standard deviation (SD) from at least 3 experiments. Statistical data were analyzed by one-way analysis of variance (ANOVA) (expressed as Mean ± SD). “*” represented the significance level (*p* < 0.05). All data were compared using SPSS 13.0 (SPSS, Chicago, IL, USA).

## 3. Results

### 3.1. The Passage of CPB Cells

**Figure 1:**
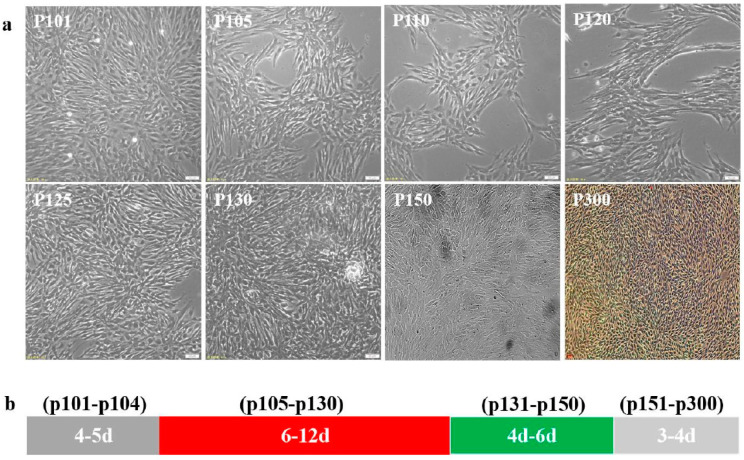
The cells state of CPB p100–p300 cells. (**a**) The cells growth state of CPB p100–p300 cells. (**b**) The interval time through a generation of p100–p104, p105–p300 and p131–p150 cells needed 4–5 d, 6–12 d, 4–6 d, 3–4 d, respectively.

The CPB p100 cells were transferred in 5% FBS L-15 for low serum acclimation. Those results showed that the low serum (5% FBS) had no significant effect on CPB cells at passage101–102, the cells began to apoptosis and the growth of cells also decreased at p103, this phenomenon continued until CPB p130, the cells were stably cultured in 5% FBS L-15 to p150, then p150 cells were continuously passaged in 8% FBS L-15to CPB p300 (Figure 1a). The passage intervals of CPB p101-p102, p103-p130, p131-p150, and p151-p300 cells were about 4–5 d, 6–12 d, 4–6 d, and 3–4 d, respectively. Those data indicated that the passage interval of CPB p300 cells were stable at 3–4 d (Figure 1b).

### 3.2. Identification of Morphological Features

**Figure 2:**
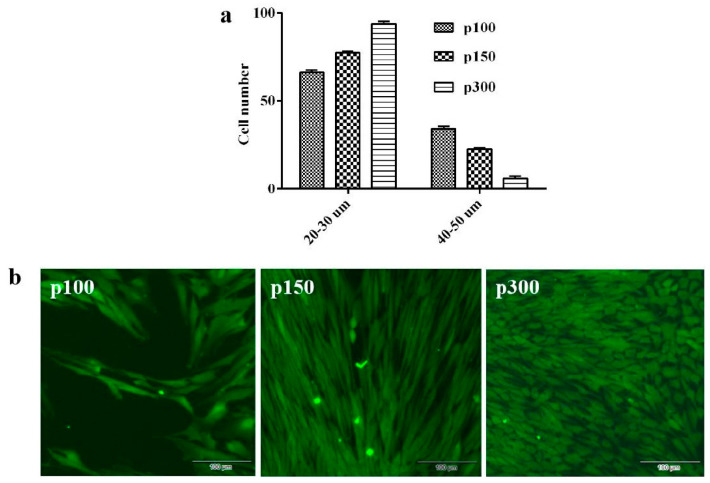
The cell morphological features of CPB p100, p150 and p300 cells. (**a**) The cell diameter of CPB p100, p150, and p300 cells. (**b**) The indirect immunofluorescence assay with cytoketatin 19 antibody of CPB p100, p150, and p300 cells showed that CPB p100, p150, and p300 cells appeared green fluoresce.

CPB p100 cells contained several different shapes, the cell morphology of CPB p150 and p300 cells were becoming more and more homogeneous. The spread diameters (20–30 µm) of CPB p100, p150 and p300 cells were about 65%, 77%, and 94%, respectively (Figure 2a). This data suggested that the small spread area of CPB p300 cells became the dominant population. An indirect immunofluorescence assay indicated that the vast majority of CPB p300 cells were epithelial cells (Figure 2b). 

### 3.3. The Growth Characteristics of CPB Cells

**Figure 3:**
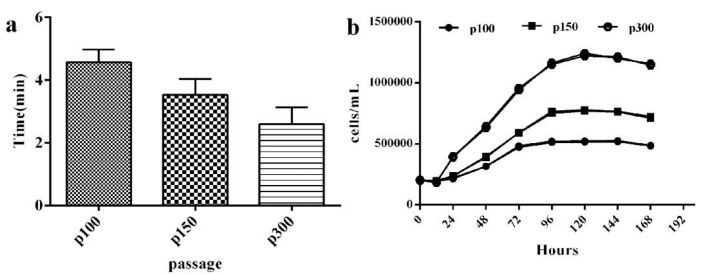
The growth characteristics of CPB p100, p150, and p300 cells. (**a**) The digestion time of CPB p100, p150, and p300 cells required 4–5 min, 3–4 min, and 2–3 min, respectively. (**b**) The growth curve of CPB p100, p150, and p300 cells.

CPB p100 cells were completely digested with 0.25% trypsin+EDTA for 4~5 min, the digestion time of CPB p150 cells was 3~4 min, However, CPB p300 cells needed to be digested for 2~3 min, which showed that the cells wall adhesion ability was becoming weaker and weaker (Figure 3a). The cell growth curves of CPB p100, p150, and p300 cells showed that the cell population doubling time of CPB p100, p150, and p300 cells were 60 h, 48 h, and 24 h, the logarithmic growth phases of CPB p100, p150, and p300 cells were 24~72 h, 24~96 h, 12~96 h, respectively (Figure 3b, Table 1). The cell multiplication times of CPB p100, p150, and p300 cells were 2.616, 3.61, and 6.2 (Figure 3b, Table 1). These results indicated that the doubling time of CPB p300 cells was 1.5 times shorter than that of CPB p100 cells, and the multiplication time of CPB p300 cells was 1.37 times higher than CPB p100 cells (Table 1). CPB p100 and p300 cells were used for follow-up comparative studies.

### 3.4. Transfection Efficiency

**Figure 4:**
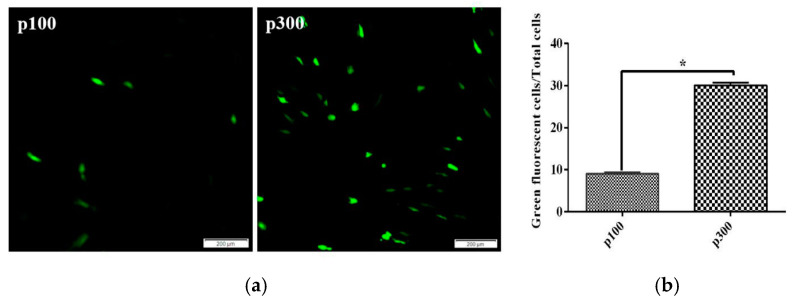
The transfection efficiency of CPB p100 and p300 cells. (**a**) The expression green fluorescent protein in CPB p100 and p300 cells, respectively. (**b**) The transfection efficiency of CPB p100 and p300 cells. “*” represented the significant difference.

We used green fluorescence protein marker to investigate their transfection efficiency. After 48 h of transfection, 10% CPB p100 cells showed green fluorescence, while 35% CPB p300 cells expressed the green fluorescent protein (Figure 4a,b). Those results indicated that the transfection efficiency of CPB p300 cells was increased by 25% compared with CPB p100 cells. 

### 3.5. Virus Susceptibility of CPB p300

**Figure 5:**
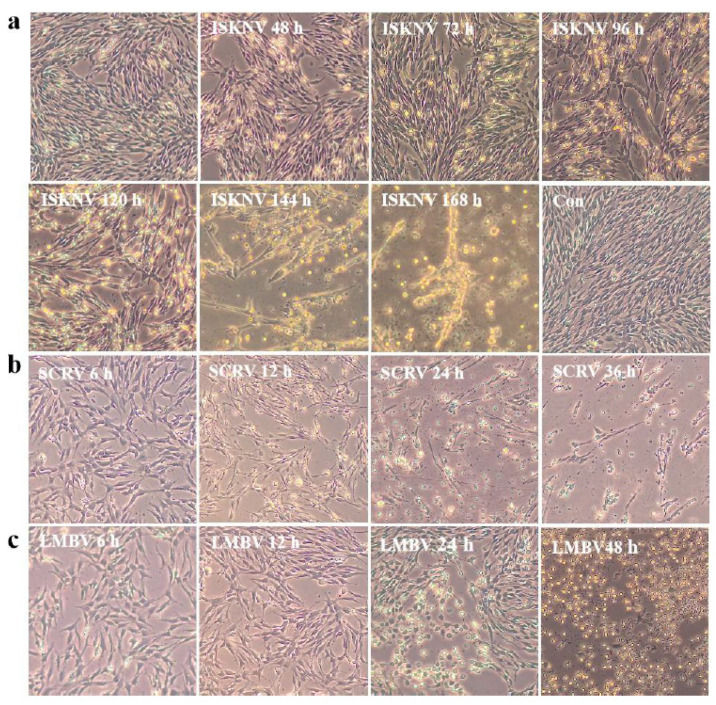
The ISKNV, SCRV ,and LMBV cytopathic effect of different time points in CPB p300 cells. (**a**) The ISKNV cytopathic effect of different time points in CPB p300 cells. (**b**) The SCRV cytopathic effect of different time points in CPB p300 cells. (**c**) The LMBV cytopathic effect of different time points in CPB p300 cells.

The CPB p300 cells were analyzed for their susceptibility to ISKNV, SCRV, and LMBV. ISKNV susceptibility: the typical cytopathic effect (CPE) of ISKNV showed cell swelling and rounding at 48 h post infection, the CPE cells were clustered and started shedding at 96 h post infection, 90% cells shed at 144 h post infection, its entire process was around 6~7 d (Figure 5a), this result indicated that the speed and progression of ISKNV CPE was relatively slow; SCRV susceptibility: the obvious expressions of CPE included increased cell diopter, rounding reduced cells and filamentous cells at 12 h post infection, CPE included rounding reduced cells and filamentous cells at 24 h, the entire cytopathic process was approximately 24~36 h (Figure 5b), the data suggested that the SCRV infection has the rapid rate and progression of CPE; LMBV susceptibility: the expressions of CPE were presented with rounding reduced cells, nuclear shrinkage, and increased cell diopter at 24 h post infection, the main presentation of CPE were cells rounding and shedding at 48 h post infection, the entire course of LMBV was about 48 h~72 h (Figure 5c), the result indicated that the speed and progression of LMBV CPE was moderate. Those results indicated that ISKNV, SCRV, and LMBV appeared at different speeds in the progression of the CPE. The cells infected with ISKNV, SCRV, and LMBV, respectively, showed corresponding typical CPE, indicating that CPB p300 was susceptible to ISKNV, SCRV, and LMBV.

### 3.6. The Optimal Conditions of Viral Multiplication in CPBp300 Cells

**Figure 6:**
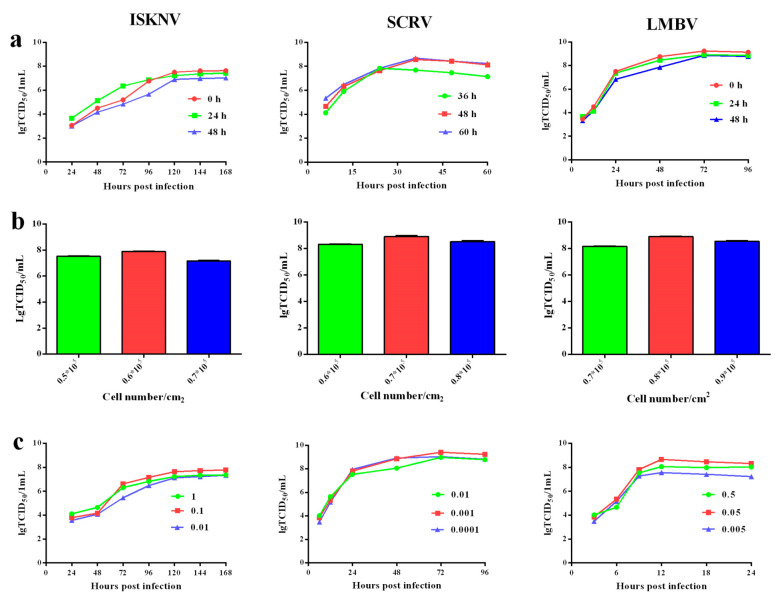
The optimal incubation time, cell density, and incubation viruses dose of ISKNV, SCRV, and LMBV multiplication in CPB p300 cells, respectively. (**a**) The optimization of ISKNV, SCRV, and LMBV incubation time was 0 h, 48 h, and 0 h, respectively. (**b**) The optimization of cell densityin ISKNV, SCRV, and LMBV multiplication was 0.6 × 10^5^ cells/cm^2^, 0.8 × 10^5^ cells/cm^2^ and 0.7 × 10^5^ cells/cm^2^. (**c**) The optimization viruses dose of ISKNV, SCRV, and ISKNV incubation was MOI = 0.1, MOI = 0.001, and MOI = 0.05, respectively.

In order to obtain higher ISKNV, SCRV, and LMBV titers, we optimized the infection time, cell concentration and virus incubation dose of ISKNV, SCRV, and LMBV multiplication. ISKNV multiplication: the viral titer of ISKNV at 0 h and 24 h of infection were higher than its infection at 48 h (Figure 6a). Considering the time cost, we chose 0 h for ISKNV infection, that is, ISKNV and cells were incubated at the same time. The virus yield of 0.6 × 10^5^ cells/cm^2^ was higher than 0.5 × 10^5^and 0.7 × 10^5^ cells/cm^2^ (Figure 6b). The viral titer of MOI = 0.1 is higher than MOI = 1 and 0.01 (Figure 6c). Those results indicated that the optimal conditions of ISKNV multiplication were simultaneous incubation, 0.6 × 10^5^ cells/cm^2^ and MOI = 0.1.

SCRV multiplication: the virus yields of SCRV at 48 and 60 h were higher than its infection at 24 h, then SCRV titer of 0.8 × 10^5^ cells/cm^2^ was relatively highest among 0.7 × 10^5^ cells/cm^2^ and 0.9 × 10^5^ cells/cm^2^ (Figure 6a,b). The virus yield of MOI = 0.01 was higher than 0.1 and 0.001 (Figure 6c). Those data suggested that the optimal conditions of SCRV multiplication were infection at 48 h, 0.8 × 10^5^ cells/cm^2^ and MOI = 0.01.

LMBV multiplication: the virus yield of LMBV at 0 h of infection was slightly higher than its infection at 24 h and 48 h (Figure 6a). The LMBV titer of 0.7 × 10^5^ cells/cm^2^was relatively higher than 0.6 × 10^5^ cells/cm^2^ and 0.8 × 10^5^ cells/cm^2^, then the viral titer of MOI = 0.05 was higher than 0.5 and 0.005 (Figure 6b,c). The optimal conditions of LMBV multiplication were simultaneous incubation, 0.7 × 10^5^ cells/cm^2^ and MOI = 0.05.

### 3.7. The Time and Economic Cost of Viral Multiplication

**Figure 7:**
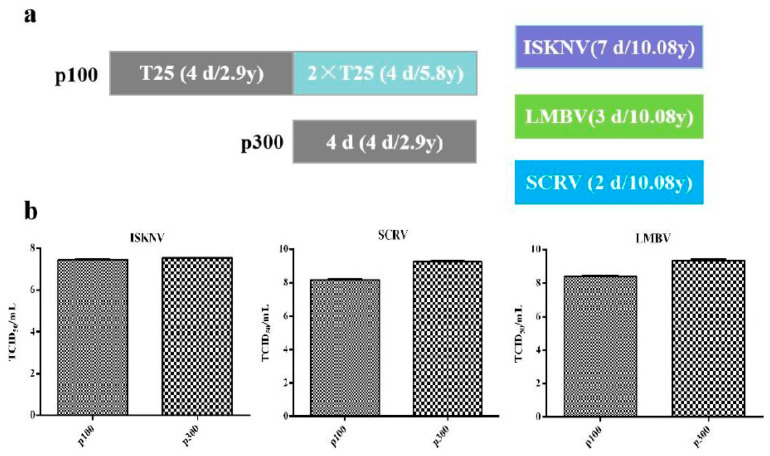
The economic and time cost of virus multiplication. (**a**) The economics and time of the production of 15 mL ISKNV, SCRV, and LMBV in CPB p100 and p300 cells. (**b**) The TCID_50_/mLof ISKNV, SCRV and LMBV in CPB p100 and p300 cells.

The CPB p100 cells from cell recovery to culture in T75 flask needs double passage and 8 days, and the cytopathic progression of ISKNV, SCRV, and LMBV were 7, 2, and 3 days, respectively. Therefore, it took 15, 11, and 10 days from cell recovery to the production of 15 mL ISKNV, SCRV and LMBV in CPB p100 cells, respectively (Figure 7a). However, CPB p300 cells from cell recovery to cell culture in T75 needs one passage and 4 days, so CPB p300 cells recovery to the production of 15 mL ISKNV, SCRV, and LMBV needed 11, 7, and 6 days, respectively (Figure 7a). The TCID_50_of LMBV and SCRV in CPB p300 cells increased 10 times than in CPB p100 cells. TCID_50_of ISKNV in CPB p300 cells was not significantly different from that in CPB p100 cells (Figure 7b). The TCID_50_/yuan of ISKNV in CPB p300 cells was 1.7 times higher than CPB p100 cells, TCID_50_/yuan of SCRV in CPB p300 cells increased 19 times than that in CPB p100 cells, the TCID_50_ of LMBV in CPB p300 cells increased 13 times than that in CPB p100 cells. The TCID_50_/d of ISKNV in CPB p300 cells was 1.87 times higher than that in CPB p100 cells, the TCID_50_/d of SCRV in p300 cells was 24 times higher than that in CPB p100 cells, the TCID_50_/d of LMBV in p100 cells was 14.4 times higher than that in CPB p100 cells (Table 2). These data indicated that the time and economic cost of ISKNV, SCRV and LMBV in CPB p300 cells were lower than CPB p100 cells.

## 4. Discussion

Largemouth bass and mandarin fish are important economic species, widely distributed in China. ISKNV, SCRV, and LMBV outbreaks frequently have caused significant economic losses to the largemouth bass and mandarin fish industry [3,24]. Vaccination is the most cost-effective measure to prevent and control fish viral diseases [25]. Compared with the aquaculture industries in developed countries, such as Europe and United States, the vaccine prevalence within the aquaculture industry is not high, either in terms of covered varieties or single species in China [26,27]. The main problem is that the vaccine varieties are few and incomplete, which is not enough to form the complete disease immune comprehensive prevention and control system. Meanwhile, the long process of developing an effective fish vaccine has reduced the farmer’s confidence for vaccine industrial application, and caused negative effects in the popularization and industrialization of fish vaccine. Cells are important substrates for production of viral vaccines. The cell synchronization and proliferation ability influence the development of vaccine production process [28]. In this study, CPB p100 cells were acclimated in low-serum with 5% FBS for 50 passage, then transferred to 8% FBS for 150 passage, we designated the passage cells as CPB p300. The growth rate and proliferation time of CPB p300 cells were significantly higher than CPB p100 cells. The size and morphology of CPB p300 cells were relatively uniform. Further studies showed that the CPB p300 cells were susceptibility to ISKNV, SCRV, and LMBV. These results suggested that the CPB p300 cells promoted the development of vaccine production process, shortened developing fish vaccine process, and enhanced the market competitiveness of these vaccines.

The production process of the fish vaccine was mainly based on the traditional rotating bottle cells production process. However, Announcement No. 1708 of the Ministry of Agriculture clearly stipulated that the GMP acceptance application of production line of veterinary cell vaccine by rotating bottle culture was suspended from 1 February 2012. The Announcement indicated that the production of fish cell vaccine must be adopted cell suspension culture technology. Cell, culture mediums, and the cell culture process are three key elements of cell suspension culture technology [29,30]. The biological features of cells determine the future market competitiveness of vaccine product [31]. The CPB cells serve as an important substrate for production viral vaccines against ISKNV, SCRV, and LMBV [16,17]. The doubling time and proliferation rate of CPB p100 cells were 72 h and 2–3 times, respectively. CPB p100 cells were not uniform in size and morphology. The heterogeneity and slow proliferation of CPB p100 cells seriously hindered the development of ISKNV, SCRV, and LMBV vaccines suspension technologies and the industrialization process of those vaccines. In order to improve the proliferation and synchronization of CPB p100 cells, CPB p100 cells were acclimated in low serum (5% FBS) L-15. The proliferation rate of CPB p300 cells was 6.2, and the doubling time was 24 h. Compared with CPB P100 cells, the proliferation rate and doubling time were increased by more than 2 times. The significant enhancement of proliferative ability reduced production material costs and time costs. The improvement synchronization of CPB p300 cells increased the quality, stability and future market competitiveness of those vaccines. 

The successful expression of foreign genes in eukaryotic cells is an important tool for studying the fine structure and regulation mechanism of the genes in cells [32]. After transfection of pEGFP, 35% cells successfully expressed green fluorescent protein. Compared to CPB p100 cells, the transfection efficiency increased by 25% [15]. This result suggested that the improvement of transfection efficiency in CPB p300 cells promoted studying the pathogenic mechanisms of ISKNV, SCRV and LMBV. 

Viruses are obligate intracellular parasites, and viral multiplication depends on cellular energy and material [33]. The virus sensitivity of cells was important for virus research and production [34]. Compared with CPB p100 cells, the ISKNV, SCRV, and LMBV sensitivity in CPB p300 cells was not different, while the SCRV and LMBV titers increased 10 times in CPB p300 cells. These data suggested that the CPB p300 cells were more suitable for viral ISKNV, SCRV, and LMBV production. The viral multiplication method can increase the virus yield and the cost-effectiveness [35,36].The speed and progression of three viruses CPE is ISKNV < LMBV < SCRV and the difference is relatively significant, which suggested that the more suitable conditions of three viruses production were different. Therefore, we optimized the infection time, cell concentration, and virus incubation dose of ISKNV, SCRV, and LMBV multiplication. Those results indicated that the optimal conditions of ISKNV, SCRV, and LMBV multiplication were simultaneous incubation, 3.0 × 10^5^ cells/cm^2^ and MOI = 0.1, simultaneous incubation, 3.5 × 10^5^ cells/cm^2^ and MOI = 0.05, and infection at 48 h, 4.0 × 10^5^ cells/cm^2^ and MOI = 0.01.

Taken together, the CPB p100 cells performed low serum acclimation in 5% FBS L-15 for 50 passages, and cultured in 8% FBS L-15 for 150 passages, and the cells were renamed CPB p300. Compared with CPB p100 cells, the size and morphology of CPB p300 cells were more uniform, and the proliferation ability of CPB p300 cells was doubled. There was no difference in ISKNV, SCRV, and LMBV sensitivity between CPB p100 and p300 cells. We optimized ISKNV, SCRV, and LMBV proliferation condition, respectively. These data showed that CPB p300 cells promoted the future competitiveness of ISKNV, SCRV, and LMBV vaccines.

## Figures and Tables

**Table 1:** The cell doubling and multiplication of CPB p100, P150, and p300 cells.

T	0 h	24 h	48 h	72 h	96 h	120 h	144 h	168 h	Doubling	Multiplication
p100	2 × 10^5^	2.17 × 10^5^	3.15 × 10^5^	4.77 × 10^5^	5.16 × 10^5^	5.19 × 10^5^	5.20 × 10^5^	4.85 × 10^5^	60 h	2.616
p150	2 × 10^5^	2.36 × 10^5^	3.90 × 10^5^	5.89 × 10^5^	7.58 × 10^5^	7.74 × 10^5^	7.63 × 10^5^	7.16 × 10^5^	48 h	3.61
p300	2 × 10^5^	3.92 × 10^5^	6.36 × 10^5^	9.48 × 10^5^	1.16 × 10^6^	1.23 × 10^6^	1.20 × 10^6^	1.15 × 10^6^	24 h	6.2

**Table 2:** The TCID_50_/y and TCID_50_/d of CPB p100 and p300 cells, respectively.

Viruses	p100(TCID_50_/y)	p300(TCID_50_/y)	p100(TCID_50_/d)	p300(TCID_50_/d)
ISKNV	(2.25 ± 0.15) × 10^7^	(4.0 ± 0.15) × 10^7^	(2.52 ± 0.35) × 10^7^	(4.73 ± 0.22) × 10^7^
SCRV	(1.17 ± 0.16) × 10^8^	(2.24 ± 0.25) × 10^9^	(2.01 ± 0.35) × 10^8^	(4.84 ± 0.51) × 10^9^
LMBV	(2.00 ± 0.15) × 10^8^	(2.64 ± 0.36) × 10^9^	(3.77 ± 0.32) × 10^8^	(5.43 ± 0.35) × 10^9^

## Data Availability

The study details can be found in the paper.
